# The Impact of COVID-19 on Patients With ADPKD

**DOI:** 10.1177/20543581211056479

**Published:** 2021-11-10

**Authors:** Meherzad Kutky, Erin Cross, Darin J. Treleaven, Ahsan Alam, Matthew B. Lanktree

**Affiliations:** 1 Division of Nephrology, Department of Medicine, McMaster University, Hamilton, ON, Canada; 2 Division of Nephrology, Department of Medicine, McGill University Health Centre, Montreal, QC, Canada; 3 St. Joseph’s Healthcare Hamilton, ON, Canada

**Keywords:** autosomal dominant polycystic kidney disease (ADPKD), COVID-19, transplantation, vaccine

## Abstract

**Purpose of review:**

Patients with autosomal dominant polycystic kidney disease (ADPKD) have kidney cysts and kidney enlargement decades before progressing to advanced chronic kidney disease (CKD), meaning patients live most of their adult life with a chronic medical condition. The coronavirus disease 2019 (COVID-19) pandemic has created common questions among patients with ADPKD. In this review, we discuss COVID-19 concerns centered around a patient with a common clinical vignette.

**Sources of information:**

We performed PubMed and Google scholar searches for English, peer-reviewed studies related to “COVID-19,” “ADPKD,” “CKD,” “tolvaptan,” “angiotensin-converting enzyme inhibitors” (ACEi), “angiotensin receptor blockers” (ARB), and “vaccination.” We also evaluated transplant data provided by the Ontario Trillium Gift of Life Network.

**Methods:**

Following an assessment of available literature, this narrative review addresses common questions of patients with ADPKD in the context of the COVID-19 pandemic.

**Key findings:**

Data regarding the risk of developing COVID-19 and the risk of adverse COVID-19 outcomes in patients with ADPKD remain limited, but patients with ADPKD with impaired estimated glomerular filtration rate (eGFR), kidney transplants, or on dialysis are likely at similar increased risk as those with generally defined CKD. We provide strategies to improve virtual care, which is likely to persist after the pandemic. Current evidence suggests ACEi, ARB, and tolvaptan treatment should be continued unless contraindicated due to severe illness. When available, and in the absence of a severe allergy, vaccination is recommended for all patients with ADPKD.

**Limitations:**

This narrative review is limited by a paucity of high-quality data on COVID-19 outcomes in patients specifically with ADPKD.

**Implications:**

Patients with ADPKD who have developed advanced CKD, require dialysis, or who have received a kidney transplant are at elevated risk of COVID-19 complications.

## What was known before

Patients with chronic kidney disease are at higher risk of hospitalization and death after COVID-19. Depending on the stage of their disease, patients with autosomal dominant polycystic kidney disease can have kidney function ranging from normal to requiring dialysis or a transplant.

## What this adds

This article provides an overview of common questions raised by patients with ADPKD and a summary of the best available data to answer them. It is believed that patients with ADPKD do not have COVID-19-associated risk greater than those with chronic kidney disease from other causes.

## Background

Patients with autosomal dominant polycystic kidney disease (ADPKD) develop innumerable bilateral kidney cysts with overall kidney enlargement and a progressive decline in kidney function, typically reaching kidney failure in the fourth to seventh decade of life. Due to its autosomal dominant inheritance, more than 70% of patients have a known family history of ADPKD^1^ and diagnosis is often made after familial cascade screening and risks stratification in a pre-symptomatic stage. Early diagnosis provides the opportunity for disease-modifying therapies to slow disease progression, but leaves many ADPKD patients with knowledge of the chronic ongoing disease process while in an asymptomatic state.

The impact of the coronavirus disease 2019 (COVID-19) pandemic on all facets of life cannot be overstated. At the time of writing this article, there have been more than 150 million confirmed COVID-19 cases with over 3 million deaths (https://www.worldometers.info/coronavirus/). Rapidly evolving evidence and clinical uncertainty have provided challenges for patients and caregivers alike. Patients with chronic conditions, such as ADPKD, have often been left with many questions specifically regarding their care. Certainly, greater attention has appropriately been directed toward patients with conditions with noted immune deficiency or requiring treatment with immune suppressant therapies. Early in the pandemic, patients with chronic kidney disease (CKD) were identified as being at a potentially higher risk for poor COVID-19 outcomes.^2,3^ Multiple online tools and chat bots have been designed to assist patients to screen for potential COVID-19 with the goal of reducing transmission.^4,5^ As more data are published regarding specific comorbidities and COVID-19 risk, we can better address the concerns of specific patient populations.

In this review, we aim to address these questions by assessing the current literature regarding the risk of developing COVID-19 and COVID-19-related complications in patients with ADPKD, and CKD more generally, stratified by estimated glomerular filtration rate (eGFR). In addition, we will evaluate the ramifications of the COVID-19 pandemic on ADPKD treatment including the use of angiotensin-converting enzyme inhibitors (ACEi), angiotensin receptor blockers (ARBs), tolvaptan, transplantation, vaccination, and the role of virtual care and telemedicine.

## Box 1. Clinical Vignette

**Figure 1.**
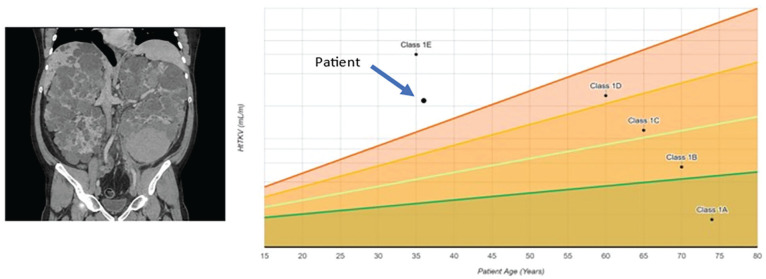
Magnetic resonance imaging and Mayo clinic risk stratification of clinical vignette.

Mr PeeKayDee is a 40-year-old man with ADPKD and hypertension. Despite having a known family history of ADPKD, he was first diagnosed 5 years ago, at age 35, when he presented to his local emergency room with gross hematuria. Ultrasound imaging confirmed the presence of numerous bilateral cysts. Further risk stratification was performed using magnetic resonance imaging measured total kidney volume which yielded a high-risk Mayo clinic imaging class of 1E (typical distribution, large high-risk kidney size, Figure 1). As such, he was started on tolvaptan, and also continues on irbesartan and amlodipine. His estimated glomerular filtration rate (eGFR) is 45 mL/min/1.73 m^2^ and has been dropping by ~5 mL/min/1.73 m^2^ per year. Mr PeeKayDee lives with his wife and 2 children. His wife, a frontline health care worker, unfortunately was exposed to SARS-CoV-2 at work and Mr PeeKayDee developed a fever shortly thereafter. He has many questions about his care:

- Am I at higher risk of COVID-19?
- Am I at higher risk of hospitalization or death if I have COVID-19?
- Should I be changing any of my medications?
- How will my care change during the COVID-19 pandemic?
- Should I get a vaccine?

## Methods

To evaluate the current evidence regarding the care of patients with ADPKD during the COVID-19 pandemic, we performed a rapid review of the literature. On February 15, 2021, we searched PubMed and Google Scholar for articles using keywords “kidney” or “renal” or “nephrology” or “polycystic kidney disease” or “autosomal dominant polycystic kidney disease” or “PKD” or “ADPKD” or “chronic kidney disease” or “CKD” or “ESRD” or “dialysis” or “GFR” or “angiotensin converting enzyme” or “ACE” or “angiotensin II receptor blocker” or “ARB” AND “Coronavirus” or “COVID” or “COVID19.” We also searched the preprint servers medRxiv and bioRxiv for material not yet through peer review. All results were then imported into Covidence systematic review software (www.covidence.org; Veritas Health Information, Melbourne, Australia) for article screening. Independent reviewers (E.C., M.K., and M.B.L.) screened texts for relevance to each section of this article. A total of 77 articles were screened with full text review in Covidence across all sections. In addition, we searched ClinicalTrials.gov for ongoing/enrolling studies as of February 23, 2021, using the search “ACE” or “ARB” and “COVID-19.” Finally, data on the rate of kidney transplantation in Ontario was obtained from the Ontario Renal Network and Trillium Gift of Life.

## Clinical Questions

### Does ADPKD Affect the Likelihood of Developing COVID-19?

A report first distributed as an unpublished preprint analysis did not show an increase in hospitalization, intensive care unit admission, intubation, or mortality following COVID-19 infection in patients with ADPKD compared with patients with other cystic kidney or cystic liver diseases.^6^ It should be noted that preprint reports have not been peer reviewed but are a growing resource for early access to research results,^7^ and this report was subsequently published.^8^ Of 709 veterans with ADPKD tested for COVID-19 in Atlanta in January 2020, 61 (8.6%) had a positive test and were compared to 9817 veterans with non-ADPKD cystic kidney or liver disease tested for COVID-19 of which 803 (8.2%) had a positive test. Symptoms in those affected with COVID-19 were no different in those with ADPKD compared with those with non-ADPKD cystic kidney or liver disease.^6^ While the data remain very limited, the biggest risk factor for COVID-19 is exposure to SARS-CoV-2, and it is unlikely that ADPKD significantly modifies this risk.

### Does ADPKD Affect My Likelihood of Hospitalization, Need for Dialysis, or Mortality If I Develop COVID-19?

Depending on their age and underlying rate of disease progression, patients with ADPKD can have the complete range of kidney function from no impairment to CKD G3 (ie, estimated glomerular filtration rate, eGFR, <60 mL/min/1.73 m^2^) to kidney failure requiring kidney replacement therapy or a kidney transplantation. The impact of immunosuppression post kidney transplant is another important consideration. In the preprint by Cui et al, patients with ADPKD were younger and had more severe kidney disease than those with non-ADPKD cystic kidney or liver disease, but after adjustment in multivariable logistic regression, the severity of kidney disease and presence of concurrent diabetes were significantly associated with hospitalization but an ADPKD diagnosis was not. The presence of CKD was also significantly associated with need for dialysis during COVID-19, but an ADPKD diagnosis was not. Between the 2 groups only 9 patients died, limiting evaluation of ADPKD as a risk factor for mortality. In a subgroup analysis of patients with CKD in Turkey, 19 patients with ADPKD were hospitalized due to COVID-19, including 2 with a kidney transplant, and 4 on dialysis, and none died during the study.^9^ While data remain quite limited, there is no evidence to suggest ADPKD increases likelihood of hospitalization, dialysis, or mortality over and above the risk conferred by the degree of CKD.

**Figure 2.**
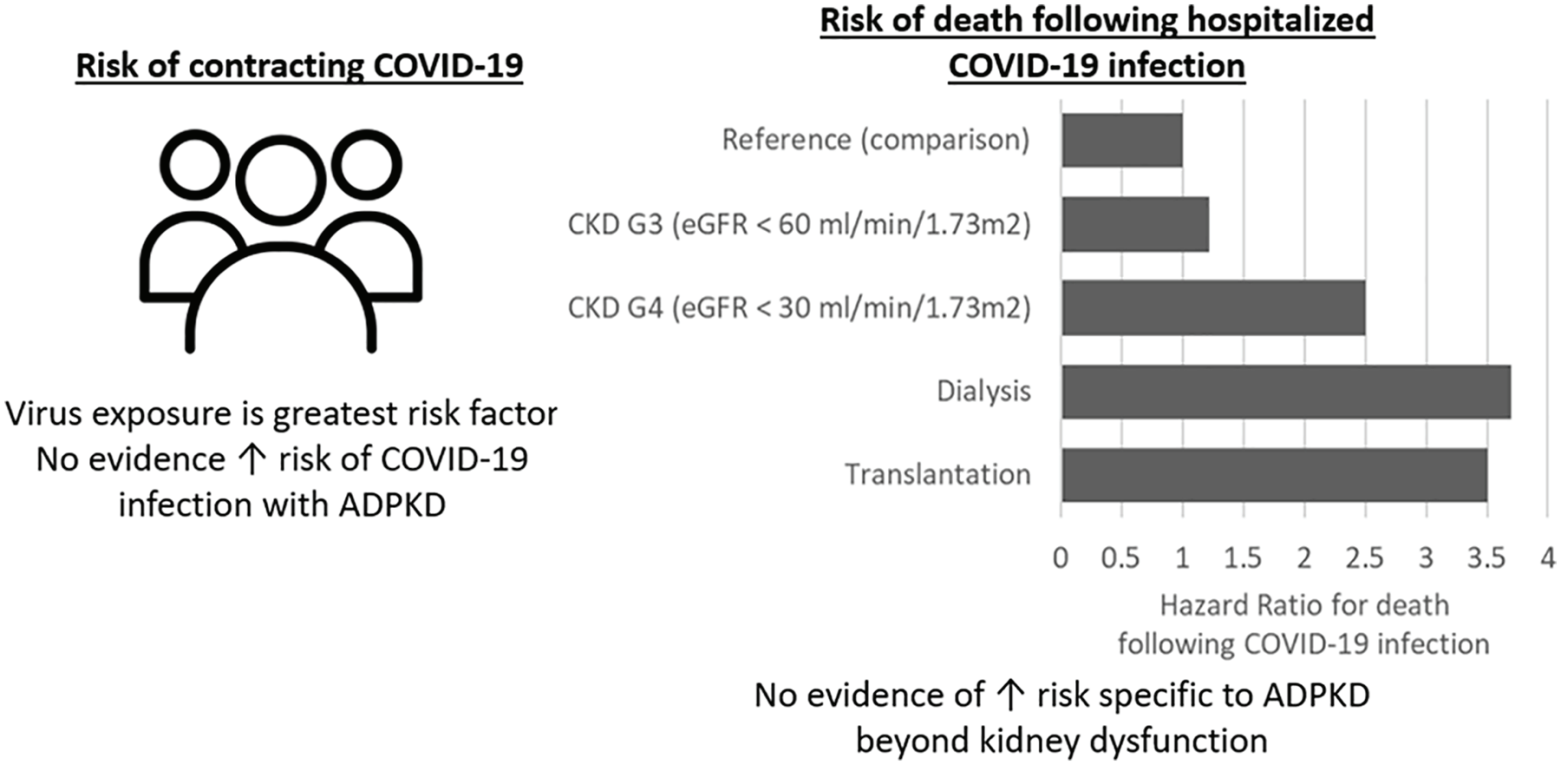
Risk of infection and risk of mortality if hospitalized with COVID-19 in patients with CKD. *Note.* Data taken from Williamson et al and Gansevoort and Hilbrands. CKD = chronic kidney disease; ADPKD = autosomal dominant polycystic kidney disease.

Large-scale population studies have sought to evaluate the relationship between CKD and COVID-19 outcomes (Figure 2). A United Kingdom population database study of 17 million patients showed increasing mortality with decreasing eGFR.^10,11^ Patients with an eGFR between 30 and 60 mL/min/1.73 m^2^ had a 22% increased risk of death, while those with eGFR below 30 mL/min/1.73 m^2^ had a 2.5-fold increased risk of death. Risk of death was further increased for patients on dialysis with a 3.7-fold greater risk, while patients with a solid organ transplant had a similar 3.5-fold greater risk.^10,11^ Data from 16 000 hospitalized COVID-19 patients with CKD (eGFR <60 mL/min/1.73 m^2^) in Mexico had a case fatality rate of 39%.^12^ A meta-analysis of 16 studies and 871 hospitalized kidney transplant patients with COVID-19 reported a pooled case fatality rate of 24%.^13^ Additional similar retrospective studies conducted in Europe, Turkey, and the United States also showed similar increased risk of mortality due to COVID-19 with decreasing renal function, kidney failure, and transplantation.^2,3,9,14,15^

### How Has COVID-19 Affected Care of Patients With ADPKD?

During the pandemic, a shift in the delivery of care for patients with ADPKD has occurred. In-person clinic visits have often been replaced with virtual care to prevent SARS-CoV-2 exposure and improve physical distancing while attempting to continue optimal care. Due to improved patient convenience, especially in tech-savvy patients, it is likely some form of virtual care will persist after the pandemic. The largest challenges for virtual ADPKD care during the pandemic are increased reliance on patients for data acquisition and health screening, such as blood pressure and weight measurements, reduced access to risk stratification (eg, medical imaging for kidney volume assessment), challenges of virtual counseling and education, and lack of physical examination.

**Figure 3.**
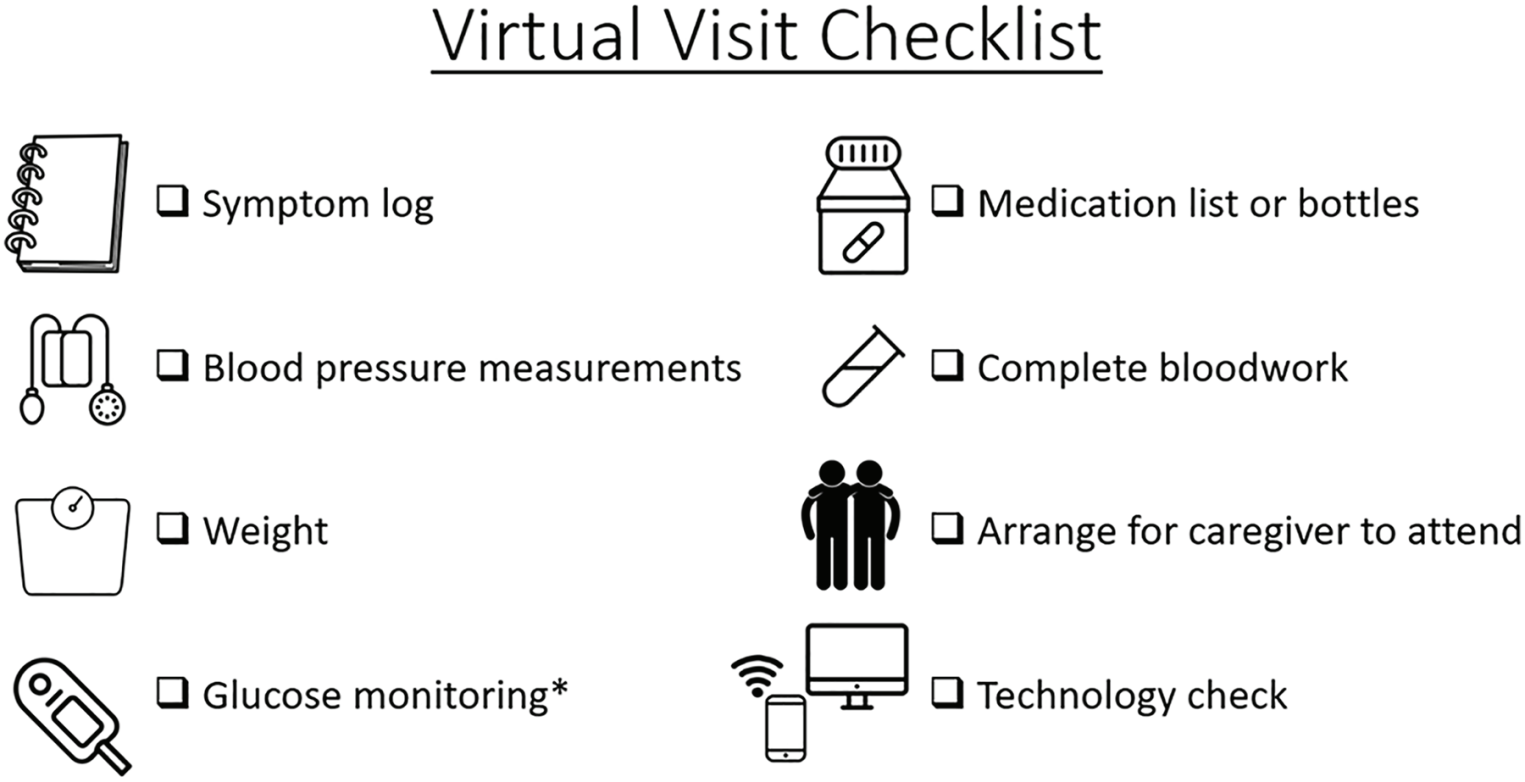
A virtual visit checklist to optimize efficiency of virtual care. *For patients with diabetes.

For in-person appointments, arranging and planning of transportation to the doctor’s is the first step for some patients and provides notice for patients to begin preparation for the visit (ie, ensuring blood pressure logs are updated). Those who choose to have caregivers and family members involved in their visit may also need notice to allow them to plan their schedule. To improve the effectiveness and efficiency of virtual ADPKD care, patients should be provided with a checklist shortly before their appointment to adequately prepare (Figure 3). Contents of a virtual visit checklist should include reminders to reflect on health status with a symptom log, serial blood pressure and weight measurements, tallying of glucose monitoring when required, collection of an updated medication list, and completion of pre-visit investigations. Ideally, patients should arrange for caregivers or supports to attend as well as a “second set of ears.” Finally, a technology check should be performed to ensure access to Wi-Fi and required video conferencing software where possible. In circumstances where some or all of this information is unavailable, the result is an ineffective virtual visit to the detriment of the patient.

While virtual visits may reduce transportation time and parking costs, and reduce likelihood of SARS-CoV-2 exposure during a pandemic, one drawback of virtual visits is that they rely heavily on the capabilities of the patient to organize their data and provide accurate measurements.^16,17^ Unfortunately, this limitation may specifically exacerbate health-related outcomes in people of disadvantaged socio-economic groups. Home health monitors, including blood pressure measurement devices, have improved to mitigate these concerns and patients should be encouraged to purchase monitors recommended by Hypertension Canada and educated on proper usage.^18^ Despite the potential for error, Hypertension Canada recommends home blood pressure monitoring as a useful predictive technique.^19^ Furthermore, patients may struggle to have bloodwork completed on time as many clinical laboratories are currently operating at reduced capacity in accordance with public health guidelines to maintain social distancing. Some lab programs have been developed to facilitate home visits for blood sample collection. Access to specific testing for ADPKD risk stratification, specifically genetic testing and magnetic resonance imaging, may have been regionally delayed due to pandemic precautions and viewed as a non-essential service. From our experience, we note that perhaps the greatest drawback of virtual visits is the loss of non-verbal communication and physical observation. Counseling and education of patients is greatly facilitated by non-verbal cues indicating confusion, comprehension, anxiety, and fear. Finally, while symptom logs and quantitative metrics such as bloodwork, weight, and blood pressure are vital for volume assessment, physical examination and observation for volume status and general well-being is necessarily inhibited in virtual visits.

### Should There Be a Change in Blood Pressure Treatment?

The question of continuing ACEi or ARB treatment is particularly pertinent to patients with ADPKD, as they are the recommended first-line treatment for the hypertension present in most patients with ADPKD, and strict blood pressure control slowed growth in total kidney volume in the HALT-PKD trial.^20^ Early in the course of the pandemic, questions were raised regarding the use and continuation of ACEi and ARB in patients with COVID-19, given the SARS-CoV-2 virus used the ACE2 receptor for the site of cell entry.^21,22^ Competing hypotheses exist that suggest either ACEi or ARB could be harmful by upregulation of the number of ACE2 receptors available for viral binding, or beneficial by decreasing the inflammation and fibrosis that leads to lung fibrosis after infection.^21^ To date, 2 published randomized trials have addressed the efficacy of ACEi/ARB use in hospitalized patients with COVID-19.

The REPLACE COVID trial was a prospective, randomized trial conducted in patients previously receiving a rennin-angiotensin system (RAS) inhibitor admitted to hospital with COVID-19. A total of 152 participants were enrolled and randomized to either continue or discontinue RAS inhibitor therapy. Participants who continued RAS inhibition had the same length of hospital stay, same length of intensive care unit stay, and same time on invasive mechanical ventilation compared to those with RAS inhibition discontinuation.^23^ BRACE CORONA randomized 740 patients with COVID-19 across 29 centers in Brazil to either discontinue or continue ACEi or ARB. The primary outcome demonstrated no difference in number of days alive and out of the hospital.^24^ It should be noted that patients on multiple antihypertensives, those with heart failure, those with reduced ejection fraction (HFrEF), or on combination ARB/neprilysin inhibitors were excluded from the study and further study is required for these sub-groups.

A recent systematic review and meta-analysis of 102 observational studies assessed the correlation of ACEi/ARB use on the likelihood of SARS-CoV-2 infection, mortality, and severe outcomes.^25^ Notably, only 49 of these were of adequate quality to include in the analysis, but Xu et al showed prior use of ACEis/ARBs was not associated with altered risk of SARS-CoV-2 infection, or significant change on severe outcomes or mortality after adjustment for confounding factors.^25^

Together, these data suggest that there is no clear evidence demonstrating that ACEi/ARBs should be discontinued in patients with COVID-19, unless for another valid clinical reason (ie, severe hypotension, hyperkalemia). This is in keeping with the current position statements from the American College of Cardiology, American Heart Association, Heart Failure Society of America, Canadian Cardiovascular Society, Canadian Heart Failure Society, and European Society of Cardiology.^26,27^

As of February 23, 2021, an additional 22 trials are registered on ClinicalTrials.gov as recruiting or enrolling to evaluate whether RAS blockers affect disease severity or mortality in patients with confirmed COVID-19. The trials differ subtly in terms of the tested intervention, doses used, patient populations, and primary outcomes. Some are examining new ACEi or ARB initiation, while other studies aim to compare discontinuation versus continuation of ACEi or ARB treatment on mortality, time in critical care, and length of hospitalization. Currently, none of the trials are evaluating patients on ARB/neprilysin inhibitor therapy. These trials will provide a more robust body of evidence regarding management of ACEi/ARB therapy in patients with COVID-19.

### Does COVID-19 Affect the Use of Tolvaptan in Patients With ADPKD?

Tolvaptan, a V2-selective vasopressin antagonist, is the only approved disease-specific treatment for ADPKD and has been shown to decrease growth in total kidney volume and slow decline in eGFR. Caveats to tolvaptan treatment include polyuria, polydipsia, and the risk of liver injury requiring regular liver enzyme monitoring.^28^ There is no proposed mechanism or evidence to suggest altered risk of SARS-CoV-2 infection or altered outcomes following COVID-19 with tolvaptan use. However, there are some challenges specific to the COVID-19 pandemic. Access to public restrooms has become restricted which can make managing polyuria difficult during essential travel. On the contrary, stay-at-home orders have created an opportunity for becoming accustomed to tolvaptan-associated polyuria while access to a washroom is easy. Mobile laboratory services to provide home blood draws have been developed, as accessing community labs can be challenging or problematic. Finally, there is no specific need to stop or hold tolvaptan if the patient remains asymptomatic, but patients should be reminded of sick day protocols and discontinue treatment with tolvaptan should they become ill.^28^

### Has COVID-19 Affected the Rate of Kidney Transplantation?

**Figure 4.**
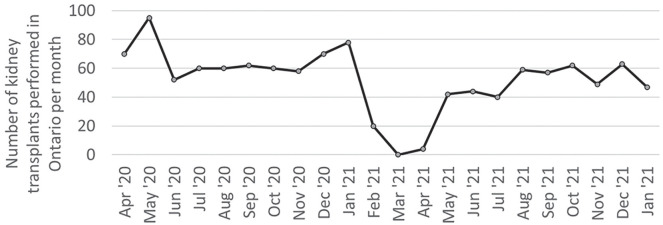
Kidney transplantation rates in Ontario decreased at the onset of the global pandemic but quickly returned to pre-pandemic rates.

We assessed the Ontario Trillium Gift of Life data to assess the rate of kidney transplantation. There was a significant decrease in the number of transplants performed at the onset of the global COVID-19 pandemic, but rates of transplantation quickly returned to pre-pandemic levels (Figure 4). Management of hospitalized kidney transplant patients with COVID-19 is beyond the scope of this review, but recommendations have been published that include supportive therapy and reduced doses of immunosuppression.^29^ The American Society of Transplantation also suggests that patients may have their cell cycle inhibitors or calcineurin inhibitors lowered or discontinued, but these decisions should be tailored to individual patients based on their illness and risk of rejection.^30^

### Should Patients With ADPKD Get a COVID-19 Vaccination?

There are no recommendations for COVID-19 vaccination that are specific to people with ADPKD. There are no limitations on vaccination based on kidney function, and for those with eGFR <30 mL/min/1.73 m^2^ vaccination is of special importance due to the greater risk of negative outcomes in the context of COVID-19.^2,3^,^30^ There are recent concerns regarding the safety of adenovirus-based vaccines (Oxford-Astra Zeneca and Janssen-Johnson & Johnson) and risk of thrombosis and vaccine-induced thrombotic thrombocytopenia.^31,32^ Despite the higher prevalence of intracranial aneurysms in people with ADPKD,^1,20^ there are no concerns regarding these vaccines specific to patients with ADPKD, and patients should be counseled to follow the advice of local public health authorities. In general, patients with ADPKD should be counseled to receive COVID-19 vaccination in accordance with local public health guidance in the absence of a previous severe allergy.^[Bibr bibr32-20543581211056479]^

## Conclusions

The COVID-19 pandemic has created challenges for patients with ADPKD and their health care providers. There is currently no evidence that patients with ADPKD are at higher risk of SARS-CoV-2 infection and exposure to the virus remains the greatest risk factor. Of those who develop COVID-19, the risk for hospitalization, intensive care admission, and death is increased in people with kidney dysfunction, but ADPKD specifically does not appear to modify risk. Patients and providers alike have grown accustomed to virtual care solutions, and it is likely to continue as a part of ADPKD care after the pandemic is over. ACEi and ARB are common medications in patients with ADPKD and should be continued unless contraindicated for acute illness. Similarly, patient risk stratification and blood test monitoring have been challenged by pandemic restrictions, but tolvaptan treatment can be continued unless patients become acutely unwell. There was an initial slowdown in kidney transplantation at the onset of the pandemic, but they have generally returned to pre-pandemic rates. Finally, patients with ADPKD should be encouraged to receive COVID-19 vaccinations in accordance with local public health guidance.
